# Rectal perforation following paclitaxel and carboplatin chemotherapy for advanced ovarian cancer: a case report and review of the literature

**DOI:** 10.1186/s13256-018-1759-z

**Published:** 2018-08-16

**Authors:** Sujen Jayakody, Danette Bianca Wright, Corrina Chiong, Mona Liu, Clare Bouffler, Toufic El-Khoury

**Affiliations:** Department of Colorectal Surgery, Westmead Hospital, Cnr Hawkesbury Road and Darcy Road, Westmead, NSW 2145 Australia

**Keywords:** Paclitaxel, Chemotherapy, Rectal perforation

## Abstract

**Background:**

Paclitaxel is a chemotherapy drug commonly used in the management of ovarian cancer. Colonic perforation is an extremely rare complication of paclitaxel administration with few case reports in the medical literature. We report a case of a patient with advanced ovarian cancer who had a rectal perforation following administration of paclitaxel. There has only been one other case report of rectal perforation in the medical literature following paclitaxel therapy.

**Case presentation:**

A 55-year-old Caucasian woman with advanced ovarian cancer awaiting elective debulking surgery for her tumor presented to our emergency department with abdominal pain, vomiting, and diarrhea. She was admitted to hospital for neoadjuvant chemotherapy and management of her systemic symptoms. She became acutely unwell following one cycle of chemotherapy with paclitaxel. A computed tomography scan of her abdomen showed typhlitis of her descending colon and a corresponding rectal perforation. Surgical intervention was deemed inappropriate as she had a heavy burden of disease and neutropenia. She died following a period of conservative management with strong intravenously administered antibiotics.

**Conclusions:**

This case highlights the importance of recognizing gastrointestinal complications following chemotherapy and the need to be aware of the possibility of bowel perforation. Prompt surgical review and intervention must be requested in patients with acute abdominal pain and persistent gastrointestinal symptoms such as diarrhea and vomiting.

## Background

Paclitaxel (Taxol®; Bristol-Myers Squibb Company, Princeton, NJ) is a biosynthetic form of the natural product taxane, which is derived from the bark of Pacific yew trees [1]. It is an anti-microtubule agent that induces the formation of stable, non-functional microtubules by enhancing tubulin polymerization. This disrupts the dynamic equilibrium within the microtubule system and halts mitosis, preventing cell replication, and causes apoptosis [2].

Paclitaxel is widely used as a first-line agent in combination with carboplatin for the management of advanced ovarian cancer [3]. Common toxicities of paclitaxel include alopecia, neutropenia, hypotension, fever, hepatotoxicity, myalgia, and peripheral neuropathy. Bowel perforation is a rare complication [4] and there are only a handful of reports of this in the literature with most occurring in the sigmoid colon [4–7]. A systematic review of the literature (MEDLINE, Embase, Google Scholar) found only one other case report of rectal perforation following combination chemotherapy with paclitaxel and carboplatin in a patient with lung cancer [8]. Table 1 shows the total number of cases found in the literature. This case report describes a case of rectal perforation following paclitaxel and carboplatin chemotherapy for advanced ovarian cancer and summarizes the literature of this rare but often fatal complication of therapy.

**Table 1:** Number of cases of bowel perforation following treatment with carboplatin and paclitaxel in the literature

Author(s) and reference number	Title of article	Type of cancer	Journal	Volume	Year	Number of cases reported
Samejima *et al*. [8]	Rectal perforation in a patient treated with combination chemotherapy for lung cancer	Lung cancer	*Gan To Kagaku Ryoho*	36(2):301–4	2009	1
de Haan and van den Berg [4]	Colonic perforation secondary to taxol therapy: an unusual presentation	Ovarian cancer	*Onkologie*	29(11):541–2	2006	1
Rose and Piver [5]	Intestinal perforation secondary to paclitaxel	Ovarian cancer	*Gynecologic Oncology*	57(2):270–2	1995	3
Seewaldt *et al*. [10]	Paclitaxel (Taxol) treatment for refractory ovarian cancer: phase II clinical trial	Ovarian cancer	*Am J Obstet Gynecol*	170(6):1666–70	1994	4
Carter and Durfee [7]	A case of bowel perforation after neoadjuvant chemotherapy for advanced epithelial ovarian cancer	Ovarian cancer	*Gynecologic Oncology*	107(3):586–9	2007	1

Table 1 indicates the number of cases of colonic perforation mentioned in the literature following treatment with paclitaxel. It indicates the type of malignancy being treated and the total number of cases detected. The journals found include non-English journals

## Case presentation

A 55-year-old Caucasian woman with a 5-month history of abdominal pain and vomiting was diagnosed as having a probable high-grade ovarian malignancy with a large volume of peritoneal disease. She had a prior ultrasound of her pelvis which demonstrated a 17 cm large irregular solid vascularized mass in her right ovary. Her comorbidities included obesity (body mass index of 33), asthma, and she had previously undergone a laparoscopic cholecystectomy. There was no significant family history. She did not smoke tobacco and she drank alcohol occasionally. She was scheduled for debulking surgery; however, she presented to our emergency department with worsening abdominal pain, vomiting, and diarrhea. A physical examination showed dry mucous membranes, capillary refill < 3 seconds, and jugular venous pressure of 4 cm. Her chest was clear on auscultation with dual heart sounds. Her abdomen was distended with generalized tenderness but no guarding or signs of peritonism. Bowel sounds were present. A computed tomography (CT) scan of her abdomen and pelvis was performed which demonstrated the large right ovarian tumor, peritoneal tumor deposits, and ascites. There was extrinsic compression of her sigmoid colon due to the tumor without radiological signs of large bowel obstruction. There were no other abnormalities of her bowel. She received intravenously administered fluids for rehydration, anti-emetics for nausea, and intravenously administered morphine for abdominal pain. She remained overnight in our emergency department for treatment. She was reassessed the following morning after resolution of her symptoms and was found to be hemodynamically stable and subsequently discharged home.

She re-presented 1 week later with similar symptoms and was admitted to hospital for further management. Following multidisciplinary discussion, she was recommended for neoadjuvant chemotherapy prior to surgical debulking. An urgent core biopsy of the mass confirmed the likely diagnosis of ovarian malignancy. This biopsy indicated a high-grade serous adenocarcinoma. The histology and CT findings were consistent with an International Federation of Gynaecology and Obstetrics (FIGO) stage III ovarian cancer. She was appropriately counselled as to the benefits and risks of chemotherapy prior to commencing treatment.

**Fig. 1:**
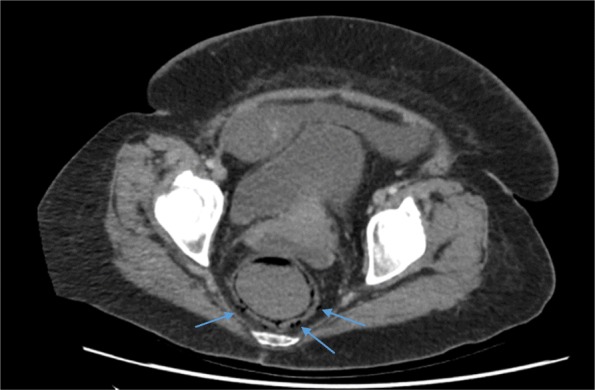
Rectal pneumatosis. Air is present in the wall of the rectum as indicated by *arrows* on coronal section of computed tomography scan of the pelvis

**Fig. 2:**
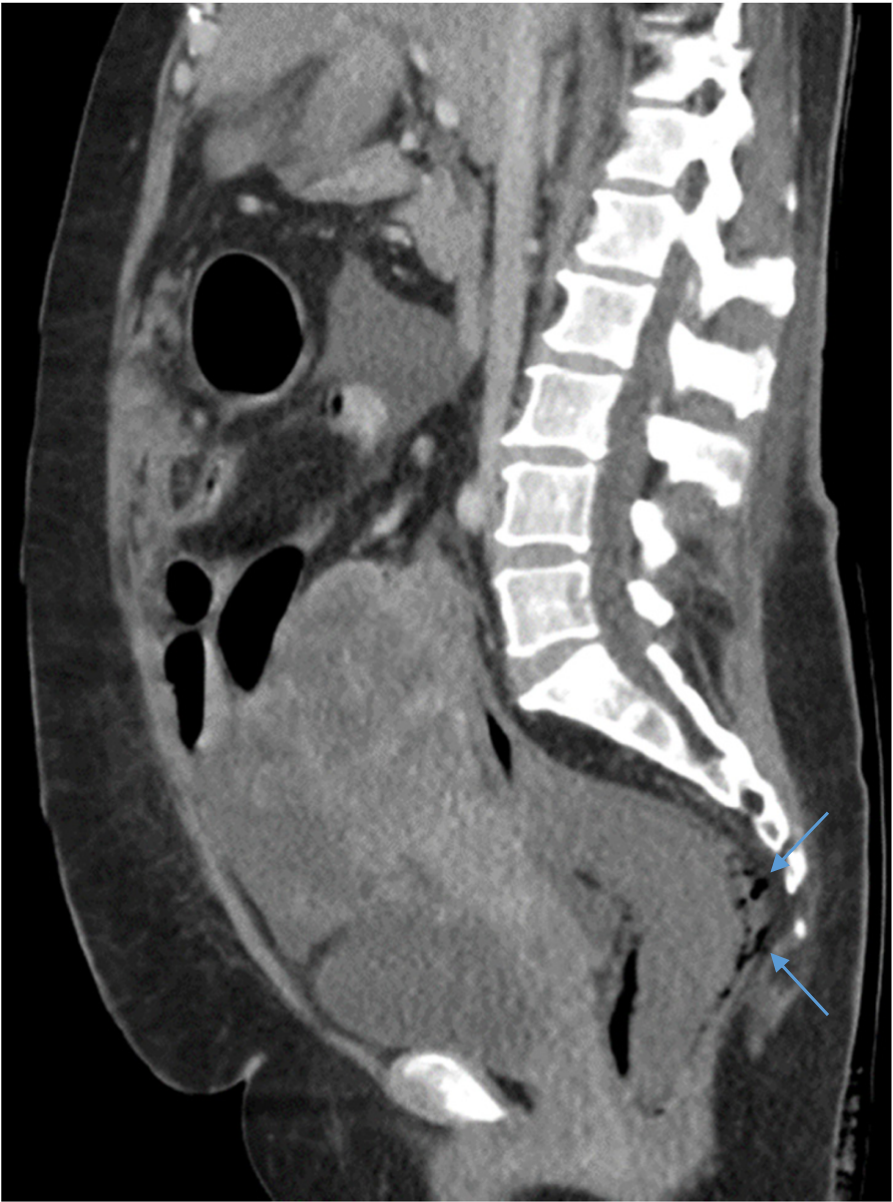
Rectal perforation. Free air is present outside the rectum as indicated by *arrows* on sagittal section of computed tomography scan of the pelvis

She was commenced on a first cycle of the commonly used platinum-based two-drug chemotherapy regime of paclitaxel and carboplatin [9]. The dose prescribed was a three weekly cycle of paclitaxel 100 mg/m^2^ and carboplatin 385 mg/body to achieve area under the curve (AUC) of 5 using the Calvert formula. The results of her pre-chemotherapy blood tests were within acceptable ranges. Specifically, her white cell count (WCC) was 10.1 × 10^9^/L (normal 4–11 × 10^9^/L) and neutrophils were 8.5 × 10^9^/L (normal 1.5–8 × 10^9^/L). She reported feeling better 1 day after chemotherapy. However, 3 days following the commencement of chemotherapy, persistent diarrhea developed. Stool cultures were negative for stool pathogens including *Clostridium difficile*. On the sixth day post-chemotherapy, she became febrile and acutely unwell with severe abdominal pain. Blood tests at the time revealed a WCC of 0.6 × 10^9^/L and neutropenia of 0.1 × 10^9^/L. A repeat CT of her abdomen and pelvis showed a thickened descending colon and rectal pneumatosis with perforation into the mesorectum (Figs. 1 and 2). Blood cultures were positive for *Escherichia coli* and meropenem was commenced. She was transferred to our intensive care unit for hemodynamic support due to septic shock. A surgical assessment was conducted but immediate surgical intervention was deemed inappropriate due to high predicted mortality. She was administered granulocyte colony-stimulating factor but her white blood cell counts did not improve and her respiratory function deteriorated. She died 9 days after the administration of the first dose of chemotherapy. An autopsy was not performed due to the wishes of our patient’s family.

## Discussion and conclusions

Gastrointestinal complications from chemotherapy range from common side effects such as vomiting and diarrhea to rarer complications such as neutropenic enterocolitis. Vomiting and diarrhea are common side effects of paclitaxel therapy. There are few case reports of neutropenic enterocolitis and reports of bowel perforation are even rarer following paclitaxel therapy.

Seewaldt *et al.* were the first to describe bowel perforation as a potential complication of paclitaxel therapy [10]. They postulated a direct toxic effect of paclitaxel causing gastrointestinal necrosis as a mechanism of action. A subsequent report by these authors reported the incidence of gastrointestinal necrosis following paclitaxel to be ~ 3% (7/238 cases) [6]. Rose and Piver reported three cases of bowel perforation following paclitaxel and also postulated a direct drug effect [5]. Of interest, in most of these cases, bowel perforation only occurred after the first cycle suggesting that paclitaxel may simply unmask a pre-existing condition [6]. Bowel perforation on subsequent cycles has, however, been reported [4, 5]. Concomitant administration of dexamethasone has also been considered a risk factor for intestinal perforation in paclitaxel therapy [4]. Tumor lysis following paclitaxel therapy also possibly contributes to the occurrence of intestinal perforation [7].

The case described here had descending colon colitis as well as a more distal perforation in her rectum both of which looked normal on earlier imaging prior to the administration of chemotherapy. It is unusual for the rectum to develop ischemia and subsequent pneumatosis due to its excellent blood supply from the inferior mesenteric, internal iliac, and median sacral arteries. The perforation in this case was not due to tumor lysis as there was no rectal invasion by the tumor. There were also no other postulated risk factors for intestinal perforation such as concurrent corticosteroid use.

It is likely that paclitaxel has several mechanisms of action. One mechanism of action of paclitaxel has a direct effect on gastrointestinal mucosa: it arrests cellular division and promotes cell death, which leads to perforation. This was suggested by Hruban and colleagues who showed the presence of gastrointestinal mitotic arrest in autopsy-derived gastrointestinal specimens following paclitaxel treatment [11]. There is also a secondary effect of paclitaxel treatment that leads to febrile neutropenia and typhlitis [12]. It is highly probable that both these effects were seen in our patient leading to neutropenic colitis in her descending colon and a simultaneous perforation of her rectum. Unfortunately, both these conditions have a high morbidity and mortality once diagnosed in the immunocompromised patient. A high index of suspicion is required early on with aggressive management in the appropriate patient to treat these complications.

Clinicians need to be aware of the risk of gastrointestinal necrosis and perforation that occurs following treatment with paclitaxel, especially on the first cycle. An analysis of these complications is important in order to identify future patients at risk of these complications. The management of gastrointestinal necrosis and perforation requires early referral to the surgical team. Surgeons need to be aware of gastrointestinal complications that occur with paclitaxel treatment and with other chemotherapy agents, including the high morbidity and mortality risks associated with these complications in what are often critically unwell patients. Treatment options need to be discussed early with the patient and in a multidisciplinary setting to ensure management is not compromised. Patients also need to have an understanding of the risks of therapy.
